# The inauguration of the English issue of the Chinese Neurosurgical Journal

**DOI:** 10.1186/s41016-015-0009-0

**Published:** 2015-08-18

**Authors:** Sheng Chen, Brandon J. Dixon, John H. Zhang

**Affiliations:** 1 Department of Neurosurgery, Second Affiliated Hospital, Zhejiang University, School of Medicine, Hangzhou, China; 2 Department of Physiology, Loma Linda University School of Medicine, Loma Linda, CA, USA; 3 Department of Anesthesiology, Neurosurgery, and Physiology, Loma Linda University School of Medicine, Loma Linda, CA, USA

## Editorial

Almost a hundred years has passed since neurosurgery became an official surgical specialty of medicine. Forged from the field of general surgery, the founders of modern neurosurgery were able to progress the field despite having rudimentary methods of anesthesia, electrocautery, understanding of post-operative hematomas, and ways to monitor intracranial pressure.

The combination of increased understanding of antiseptic techniques, advancements in surgical technologies, and early experimental successes lead to the formation of the first organized group called the Society of Neurological Surgeons in the United States in 1919. Later leading to the formation of other American societies and groups, such as The Harvey Cushing Society in 1932 [1]. On the international front the promotion of neurosurgery was also accelerating during this time. The first journal solely devoted to neurosurgery was established in Berlin in 1936 [2]. Also around this time the *Journal of Neurosurgery* was created in 1944 by the American Association of Neurological Surgeons receiving submissions from all over the world [3].

Today there are nearly two hundred neurosurgery related journals around the world and more than ten neurosurgery related journals in China [4]. Although there are many international neurosurgery journals, there has never been an English language based journal originating from China. Unfortunately, this often hindered the exchange of original neurosurgical research and information from China to the rest of the world. Since there are more than ten thousand practicing Chinese neurosurgeons, there is a great need for scientific communication in the Chinese neurosurgery community.

Professor Jizong Zhao, the first Editor-in-Chief, is to be commended for forward thinking and assisting in the courageous decision to provide a true international journal. The *Chinese Neurosurgical Journal* aims to publish work in all areas of neurosurgery from neurosurgeons and neurosurgical researchers not only from China, but also from physicians and scientists from all over the world. The journal also focuses on clinical practice and techniques, including clinical and basic science research studies, book reviews, case reports, commentaries, letters to the editor, and reviews. We believe this journal will serve the global scientific community as an excellent vessel to exchange information between Chinese neurosurgeons and neurosurgeons from other countries. We also expect that this new English version of the *Chinese Neurosurgical Journal* will enjoy success under the leadership of Professor Jizhong Zhao, and will greatly contribute to neurosurgical research worldwide.
